# Subclinical Cardiac Dysfunction Is Associated With Extracardiac Organ Damages

**DOI:** 10.3389/fmed.2018.00323

**Published:** 2018-11-20

**Authors:** Aymeric Menet, Brigitte Ranque, Ibrahima Bara Diop, Samuel Kingue, Roland N'guetta, Mamadou Diarra, Dapa Diallo, Saliou Diop, Ibrahima Diagne, Ibrahima Sanogo, David Chelo, Guillaume Wamba, Indou Deme-Ly, Blaise Felix Faye, Moussa Seck, Aissata Tolo, Kouakou Boidy, Gustave Koffi, Eli Cochise Abough, Cheick Oumar Diakite, Youssouf Traore, Gaëlle Legueun, Ismael Kamara, Lucile Offredo, Sylvestre Marechaux, Mariana Mirabel, Xavier Jouven

**Affiliations:** ^1^Cardiology unit, Groupement des Hôpitaux de L'université Catholique de Lille, Université Catholique de Lille, Lille, France; ^2^Internal Medicine Unit, Hôpital Européen Georges Pompidou, Assistance Publique des Hôpitaux de Paris and UMR_S970, Universite Paris Descartes, Inserm, Paris, France; ^3^Cardiology Unit, CHU de Fann, Dakar, Senegal; ^4^Cardiology Unit, Hôpital Général, Yaoundé, Cameroon; ^5^Institut de Cardiologie, Abidjan, Ivory Coast; ^6^Cardiology Unit, Centre Gynéco-obstétrique, Bamako, Mali; ^7^Centre de Recherche et Lutte contre la Drépanocytose, Bamako, Mali; ^8^Centre National de Transfusion Sanguine, Dakar, Senegal; ^9^Pediatrics Unit, Centre Hospitalier National d'Enfants Albert Royer de Dakar, Université Cheikh Anta Diop, Dakar, Senegal; ^10^Hematology Unit, CHU de Yopougon, Abidjan, Ivory Coast; ^11^Cardiology Unit, Fondation Mère Enfant Chantal Biya, Yaoundé, Cameroon; ^12^Pediatrics Unit, Centre Hospitalier d'Essos, Yaoundé, Cameroon; ^13^Cardiology Unit, Libreville, Gabon; ^14^Centre de Recherche et Lutte contre la Drépanocytose, Bamako, Mali; ^15^UMR_S970, Université Paris Descartes, Inserm, Paris, France; ^16^Cardiology Department, Hôpital Européen Georges Pompidou, Assistance Publique des Hôpitaux de Paris and UMR_S970, Université Paris Descartes, Inserm, Paris, France

**Keywords:** sickle cell disease, heart failure, hemolytic anemia, cardiac remodeling, global health

## Abstract

**Background:** Several studies conducted in America or Europe have described major cardiac remodeling and diastolic dysfunction in patients with sickle cell disease (SCD). We aimed at assessing cardiac involvement in SCD in sub-Saharan Africa where SCD is the most prevalent.

**Methods:** In Cameroon, Mali and Senegal, SCD patients and healthy controls of the CADRE study underwent transthoracic echocardiography if aged ≥10 years. The comparison of clinical and echocardiographic features between patients and controls, and the associations between echocardiographic features and the vascular complications of SCD were assessed.

**Results:** 612 SCD patients (483 SS or Sβ^0^, 99 SC, and 19 Sβ^+^) and 149 controls were included. The prevalence of dyspnea and congestive heart failure was low and did not differ significantly between patients and controls. While left ventricular ejection fraction did not differ between controls and patients, left and right cardiac chambers were homogeneously more dilated and hypertrophic in patients compared to controls and systemic vascular resistances were lower (*p* < 0.001 for all comparisons). Three hundred and forty nine SCD patients had extra-cardiac organ damages (stroke, leg ulcer, priapism, microalbuminuria or osteonecrosis). Increased left ventricular mass index, cardiac dilatation, cardiac output, and decreased systemic vascular resistances were associated with a history of at least one SCD-related organ damage after adjustment for confounders.

**Conclusions:** Cardiac dilatation, cardiac output, left ventricular hypertrophy, and systemic vascular resistance are associated with extracardiac SCD complications in patients from sub-Saharan Africa despite a low prevalence of clinical heart failure. The prognostic value of cardiac subclinical involvement in SCD patients deserves further studies.

**Graphical Abstract:**
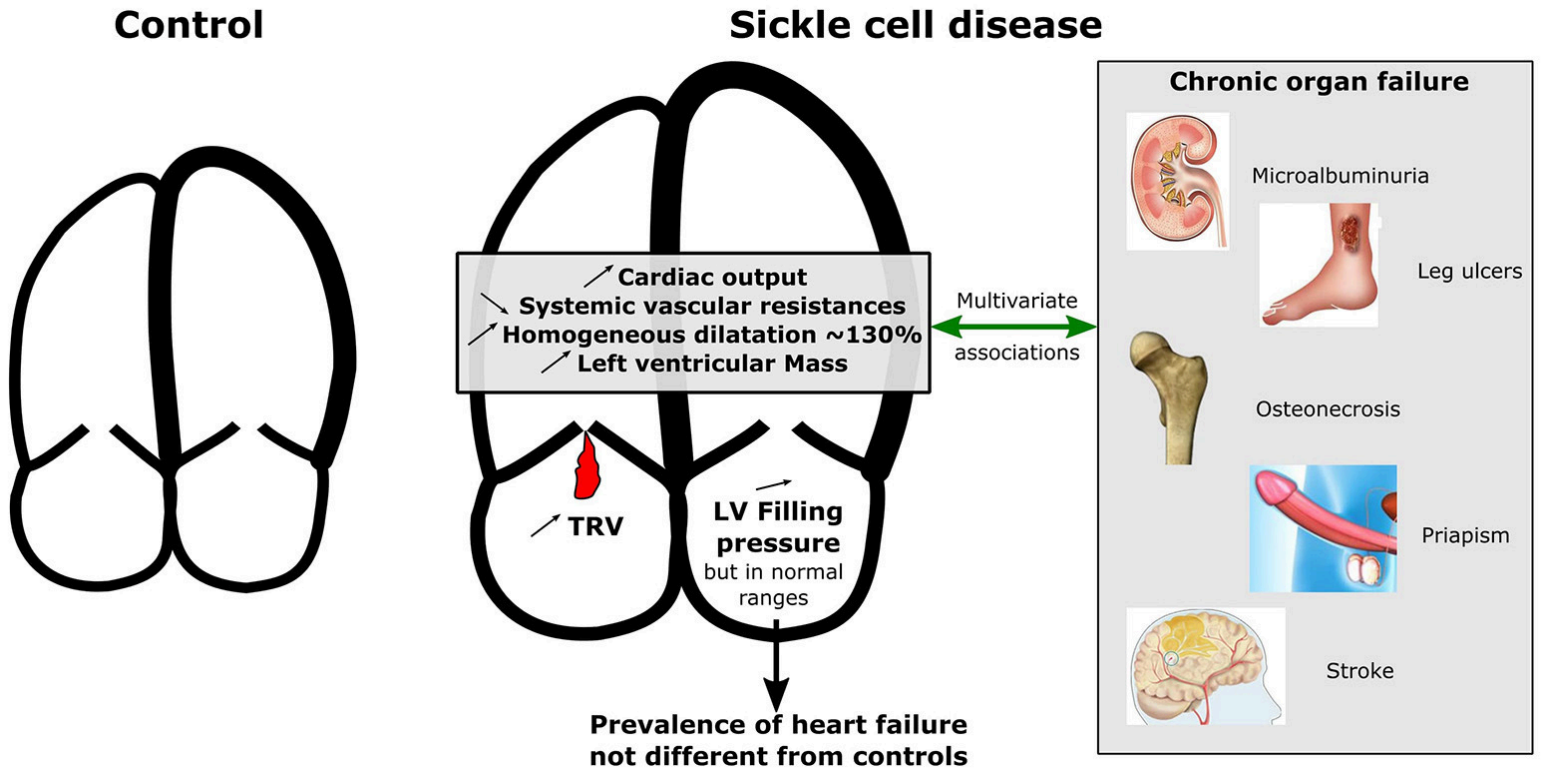
Graphical comparison for cardiac characteristics between SCD patients and controls and multivariate associations with chronic organ failure. While left ventricular ejection fraction did not differ between controls and patients, left and right cardiac chambers were homogeneously more dilated and hypertrophic in patients compared to controls and systemic vascular resistances were lower. Increased left ventricularmass index, cardiac dilatation, cardiac output, and decreased systemic vascular resistances were associated with a history of at least one SCD-related organ damage after adjustment for confounders. TRV, Tricuspid regurgitant velocity; LV, Left ventricle.

## Introduction

Sickle cell disease (SCD) is a hemoglobin genetic disorder responsible for deep chronic hemolytic anemia, recurrent acute vaso-occlusive crises, and chronic vasculopathy leading to multiple organ failure such as glomerulopathy, osteonecrosis, leg ulcers, stroke, pulmonary hypertension, and priapism. Both chronic anemia and myocardial ischemia are known to lead to heart failure in the general population (1). Major cardiac remodeling (2, 3), diastolic dysfunction and myocardial ischemia (4–6) have been described in SCD. The overwhelming majority of the data are based on reports from North America and Europe. Thus, very little is known about the prevalence of cardiac abnormalities in African SCD patients. Moreover, it is unknown whether these abnormalities are associated with other SCD-related organ damage. The CADRE (Coeur Artères et DREpanocytose, i.e., Heart Arteries and Sickle cell disease) study is a large multinational cohort of SCD patients, taking place in 5 sub-Saharan African countries. We analyzed the clinical and echocardiographic data of the patients included in the CADRE study to assess the frequency of SCD-related cardiac abnormalities in sub-Saharan Africa and looked for associations between cardiac involvement and other SCD-related organ damage.

## Materials and methods

### Settings and patients' recruitment

The CADRE study is a multinational observational cohort that includes 3,747 SCD patients aged 3 years and older and 950 healthy controls matched by age and country, recruited through outpatient clinics in Yaoundé and Douala (Cameroon), Dakar (Senegal), Abidjan (Côte d'Ivoire), Franceville (Gabon) and Bamako (Mali). The overall aim of CADRE is to provide descriptive and prognostic data in SCD patients living in sub-Saharan Africa, especially regarding the cardiovascular complications of SCD. The objective of the present study is to determine the prevalence of cardiac involvement in African SCD patients and attempt to identify factors associated with adverse outcomes. The CADRE protocol was approved by the relevant national ethics committee in each of the participating countries: Côte d'Ivoire (N043/MSLS/CNER-dkn), Mali (N18 MS-SG-CNESS/2011), Senegal (N12/40 MSAS/DS/CNERS), Cameroon (N016-CNE-SE-2011), and Gabon (N023-CNER-GB_2011). The patients were recruited from February 2011 to December 2013. A detailed protocol of the study is available elsewhere (7).

### Inclusion criteria

Three centers of the CADRE study (Yaoundé, Dakar and Bamako) participated to the echocardiographic study. These centers were selected based on the possibility to collaborate with a senior cardiologist who met the requested expertise in cardio-echography practice. In these centers, all SCD patients aged 10 or more attending the participating outpatient clinics were invited to participate in the present study. Patients (or their parents) were required to sign an informed consent form. Controls were either healthy voluntary parents or siblings of SCD patients, or healthy voluntary members of the center's medical or nursing staff.

### Data collection

The medical visits were performed at a steady state defined as the absence of any vaso-occlusive crisis (VOC) for the last 15 days, absence of fever or infectious disease for the last 8 days and absence of transfusion for the last 2 months. We recorded medical history, including the main complications of SCD and physical examination findings on standardized forms. Dyspnea was assessed by the New York Heart Association (NYHA) classification. Congestive heart failure was defined by the combination of NHYA class ≥ II and congestion or a history of congestion. Physical activity was considered if there was more than 30 min of walking 5 times per week or 20 min of physical activity reaching the threshold of breathlessness 3 times a week. Laboratory tests included alkaline hemoglobin electrophoresis and/or high-performance liquid chromatography to confirm the SCD phenotype (if not already available), complete blood counts, reticulocyte counts, bilirubin and lactate dehydrogenase (LDH) serum levels, serum creatinine, urine creatinine and quantitative albuminuria. Estimated glomerular filtration rate (eGFR) was calculated using the CKD-EPI formula without adjusting for African ethnicity for adults (8) and the Schwartz formula for children younger than 15 years old (9).

### Echocardiography

Echocardiographies were performed by a single experienced cardiologist in each center. Each cardiologist has been trained in Europe and on site by the first author to ensure good quality and reproducibility of the measures. Patients and controls were scanned by the same machine in each country. Respectively, the Sonosite Micromaxx (Sonosite, Bothell, USA), the Philips HP Sonos 5500 (Philips, Andover, Massachusetts) and the Vivid E7 commercial (GE Medical Systems, Horten, Norway) ultrasound scanner was used. We therefore adjusted for the country to limit the measurement bias. The following anatomic measurements were recorded according to the recommendation of the American Society of Echocardiography (10): septal wall, posterior wall thickness, and left ventricular (LV) minor axis dimension at end-diastole and end-systole were obtained with the use of M-mode recordings obtained at a sweep speed of 100 mm/s or with parasternal long axis view 2D imaging. LV mass was calculated using the Devereux's formula (0.8 (1.04 [septal + posterior wall + LV telediastolic diameter]3—[LV telediastolic diameter]3) + 0.6) and indexed for the body surface area. Hypertrophy was considered if indexed left ventricular mass was higher than 95 g/m^2^ in women and 105 g/m^2^ in men. Relative wall thickness was the ratio of the septum thickness plus posterior wall thickness divided by the left ventricular end diastolic diameter. Concentric hypertrophy was considered if relative wall thickness was strictly higher than 0.42. LV volume was measured by surfacing the LV from the apical four-chamber view. LV systolic ejection fraction was calculated by the Simpson's method from the apical four-chamber view. Cardiac output index was calculated as the product of the forward stroke volume (time-velocity integral x D^2^/4 x π, D being the diameter of the LV outflow tract) and heart rate divided by body surface area. Cardiac work index was calculated as the product of mean arterial pressure and cardiac output index. Systemic vascular resistances were calculated as the subtraction of estimated right atrial pressure to mean arterial pressure divided by cardiac output. Atrial surfaces were measured at ventricular end-systole in the apical four-chamber view and were indexed to body surface area. Pulsed-wave Doppler was performed in the apical 4-chamber view at end-expiration with a 3-mm sample volume to obtain mitral inflow velocities (E and A waves).(11) Pulsed-wave tissue Doppler imaging was sequentially performed to acquire lateral and septal mitral annular velocities (E'l and E's waves), and both velocities were averaged to calculate the E/E' ratio (which correlates reasonably well with LV end-diastolic pressure) as previously described (12). E/E' ratio <8 and E/E' ≥13 were considered as low and high LV filling pressures, respectively. Diastolic dysfunction was considered if E'l <10 cm/s or E's <8 cm/s (11) and was classified in three grades using E/A ratio, E wave deceleration time and E/E' ratio as previously described (11). The tricuspid regurgitation pressure gradient and the trans-pulmonary pressure gradient was recorded from any view with continuous-wave Doppler imaging with the modified Bernouilli equation (4 × V^2^), where V is the peak tricuspid regurgitation velocity or the end diastole pulmonary regurgitation. Estimated pulmonary pressures were obtained when tricuspid regurgitation and/or pulmonary regurgitation jets were available, as recommended. Pulmonary vascular resistances were calculated as the subtraction of estimated right atrial pressure to mean pulmonary arterial pressure divided by cardiac output as left and right atrium pressure are very similar. Because right heart catheterization was not an option, a tricuspid regurgitant jet velocity (TRV) strictly above 3 m/s was used as a surrogate marker of pulmonary hypertension (13, 14).

### Statistical methods

Continuous data are presented as median (25–75th percentiles). Qualitative data are presented as absolute values and percentages. Comparison between patients and controls were performed using multilevel logistic regression with country as a nested variable (to control for potential inter-center variability) adjusted for age and sex.

To investigate the association of cardiac measurements with the SCD complications we performed separated regressions in the SCD population, including clinical complications as the explained variable and cardiac measurements as explanatory variables. SCD-related organ damage was defined by the history of at least one of the following complications: microalbuminuria, osteonecrosis, leg ulcer, stroke, or priapism. We used multilevel logistic regression adjusted for age, mean BP, heart rate, hemoglobin, SCD phenotypic group, and country. Continuous variables were scaled in order to make the Odds Ratios comparable. A 2-tailed *P* value of < 0.05 was considered to be statistically significant. All analyses were performed using R software (version 3.1.0).

## Results

### Patients' and controls' baseline characteristics (Table 1)

Six hundred and twelve SCD patients (483 SS or Sβ0, 99 SC and 19 Sβ+) and 149 healthy controls underwent echocardiography. Their median (Q1;Q3) age was respectively 23 [18;30] and 26 [22;33] years old. Clinical and biological SCD features are described in Table 1 and were significantly different between the SCD phenotypic groups: SS and Sβ0 patients had a lower hemoglobin level, higher hemolysis markers, leucocyte and platelet counts and glomerular filtration rate, more frequent history of leg ulcers, more frequent micro-albuminuria (*p* < 0.05 for all comparisons). A history of at least one organ damage was present in 66% of SS and Sβ0 patients, 48% of SC, and 56% of Sβ+ patients (*p* = 0.001). Noteworthy, <1% of patients were treated with hydroxyurea or iterative blood transfusions. The prevalence of participants with NYHA class III or IV dyspnea was 2 % in patients and 0 % in controls (*p* = 0.921). Likewise, the prevalence of congestive heart failure was similar in patients and in controls (0.5 vs. 0% *p* = 0.999). There was no difference between SS/Sβ0, SC or Sβ+ patients for the prevalence of NYHA III/IV or congestive heart failure. Heart rate was significantly higher in patients than in controls and higher in SS-Sβ0 than in SC or Sβ+ patients (*p* = 0.001). Mean blood pressure (BP) was significantly lower in patients than in controls (*p* < 0.001) and lower in SS-Sβ0 than in SC or Sβ+ patients, even after adjustment for age (*p* < 0.001). Noteworthy, in the entire CADRE cohort including 3,627 patients with SCD (median age: 15 years old) and 943 healthy controls (median age: 18 years old), the prevalence of NYHA class III/IV and the prevalence of clinical heart failure did not differ in patients and in controls (respectively 1.6 vs. 0.7 %, *p* = 0.127 and 0.1 vs. 0% *p* = 0.998).

**Table 1:** Demographic and clinical data of patients with sickle cell disease and controls.

	**SCD patients**	**Controls**	**Patients vs. controls**	**Sβ+**	**SC**	**SS/Sβ0**	**SC vs.Sβ+ vs.SS/Sβ0**
	***N** =* **612**	***N** =* **149**	***P*** value^*^	***N** =* **19**	***N** =* **99**	***N** =* **483**	***P*** value^*^
**GENERAL CHARACTERISTICS**							
Age (mg/g)	23 [18;30]	26 [22;33]	<0.001	22 [17;27]	24 [20;34]	23 [18;30]	0.177
Male gender	262 (43%)	58 (39%)	0.644	7 (37%)	38 (38%)	213 (44%)	0.499
Body mass index (kg/m2)	19 [17;22]	22 [20;25]	<0.001	18 [17;23]	21 [19;24]	19 [17;21]	<0.001
Country			0.209				<0.001
Cameroun	204 (33%)	47 (32%)		2 (10%)	0 (0%)	191 (40%)	
Mali	240 (39%)	49 (33%)		14 (74%)	91 (92%)	135 (28%)	
Senegal	168 (28%)	53 (36%)		3 (16%)	8 (8%)	157 (32%)	
Smoker	22 (4%)	7 (5%)	0.695	0 (0%)	4 (4%)	18 (4%)	0.892
Physical activity	22 (4%)	15 (10%)	0.002	0 (0%)	2 (2%)	19 (4%)	0.775
Diabetes mellitus	2 (1%)	4 (4%)	0.044	0 (0%)	0 (0%)	2 (1%)	0.885
LDH level (IU/l)	694 [430;1026]	510 [437;634]	0.017	380 [313;485]	368 [292;500]	886 [636;1164]	<0.001
Bilirubin level (mg/l) ^****^	30 [22;42]	4 [2;6]	<0.001	35 [31;38]	NA	30 [22;42]	0.642
Glomerular filtration rate (ml/min)	124 [102;159]	94 [81;117]	<0.001	105 [84.1;140]	104 [86.7;127]	130 [106;164]	<0.001
**HISTORY OF COMPLICATIONS**							
NYHA 3-4	4 (2%)	0 (0%)	0.921	0 (0%)	0 (0%)	4 (2%)	1.000
History of congestive heart failure	3 (0.5%)	0 (0%)	0.999	0 (0%)	0 (0%)	3 (0.6%)	1.000
At least one VOC >48h in the previous year	4 (67%)	NA	NA	16 (84%)	81 (83%)	346 (72%)	0.064
Acute chest syndrome, lifetime	141 (24%)	NA	NA	2 (11%)	4 (4%)	133 (29%)	<0.001
Stroke, lifetime	13 (2%)	NA	NA	0 (0.00%)	2 (2%)	11 (2%)	1.000
Leg ulcer, lifetime	97 (16%)	NA	NA	1 (5%)	6 (6%)	89 (18%)	0.002
Priapism, lifetime	53 (20%)	NA	NA	2 (11%)	9 (9%)	42 (9%)	0.860
Osteonecrosis, lifetime	110 (18%)	NA	NA	3 (16%)	15 (15%)	90 (19%)	0.719
Microalbuminuria^**^	210 (40%)	25 (19%)	<0.001	6 (38%)	16 (20%)	183 (43%)	<0.001
Chronic organ complication^***^	359 (66%)	NA	NA	10 (56%)	40 (48%)	293 (66%)	0.005
**HEMODYNAMIC PARAMETERS**							
Heart rate (beat/min)	73 [66;81]	68 [61;77]	0.001	68 [61;75]	72 [65;81]	73 [66;81]	0.030
Mean blood pressure (mmHg)	79 [75;85]	85 [79;94]	<0.001	83 [79;91]	82 [78;88]	79 [74;84]	<0.001
Systolic blood pressure (mmHg)	113 [107;120]	119 [109;129]	<0.001	116 [111;121]	114 [109;121]	112 [107;119]	0.078
Diastolic blood pressure (mmHg)	62 [58;69]	68 [64;76]	<0.001	70 [61;74]	65 [61;72]	62 [57;67]	<0.001

*Quantitative value are expressed in median [Q1;Q3] and qualitative data are expressed in n (%)*.

* *p value adjusted for age and sex with country as a nested variable*.

** *microalbuminuria was defined by a urine albumin/creatinine ratio >30 mg/g*.

*** *Chronic organ complications include stroke, leg ulcer, priapism, osteonecrosis and microalbuminuria. Complications were all documented in 556 patients*.

**** *Bilirubin was not assessed in Mali*.

### Echocardiographic parameters (Table 2)

#### Cardiac morphology

The patients and controls were young with a low body mass index, therefore no patient was excluded for technical issue. The four cardiac chambers were homogeneously dilated in SCD patients (Table 2). Left ventricular mass index (99 [78;122] vs. 68 [57;86] g/m^2^, *p* < 0.001), cardiac output index (3.6 [2.9;4.2] vs. 2.3 [2.0;2.9] L/min/m^2^, *p* < 0.001) and cardiac work index (277 [229;337] vs. 209 [165;254] L.mmHg/min/m^2^, *p* < 0.001) were higher in SCD patients than in controls. Fifty percent of the patients (57% of SS/ Sβ0 patients and 18% of SC patients) had cardiac hypertrophy vs. 10% in the control group *p* < 0.001. The hypertrophy was eccentric in 62% and concentric in 38% of the SCD patients. Left ventricular mass index and cardiac output were negatively correlated with hemoglobin level and the correlation was particularly strong for patients with a hemoglobin level inferior to of 9 g/dL (Figure 1).

**Table 2:** Echo-Doppler data in 761 subjects with echocardiography.

	**SCD patients**	**Controls**	**Patients vs.controls**	**Sβ+**	**SC**	**SS/Sβ0**	**SC vs.Sβ+ vs.SS/Sβ0**
	***N** =* **612**	***N** =* **149**	***P*** value^**^	***N** =* **19**	***N** =* **99**	***N** =* **483**	***P*** value^**^
Cardiac output index (L/m2)	3.6 [2.9; 4.2]	2.3 [2.0; 2.9]	<0.001	2.8 [2.2; 3.5]	3.0 [2.3; 3.7]	3.7 [3.1; 4.3]	<0.001
Systemic vascular resistances (WU)	14 [11; 17]	20 [17; 24]	<0.001	16 [14; 24]	17 [13; 21]	13 [11; 16]	<0.001
LV ejection fraction (SD, %)	67 [61; 73]	66 [60; 71]	0.158	69 [66; 75]	70 [64; 76]	66 [60; 72]	0.742
LV ejection fraction ≤ 45%	4 (0,7%)	1 (0,7%)	0.796	0 (0%)	0 (0%)	4 (0.8%)	1.000
indexed LV end diastolic diameter (mm/m^2^)	31 [28; 34]	25 [23; 28]	<0.001	30 [26; 32]	28 [25; 30]	31 [29; 35]	<0.001
indexed LV end-diastolic volume (mL/m2)	63 [54; 74]	43 [36; 51]	<0.001	63 [51; 71]	56 [45; 65]	65 [56; 77]	<0.001
Indexed LA surface (cm^2^/m^2^)	12 [9; 14]	8 [7; 10]	<0.001	9 [8; 11]	8 [7; 10]	12 [10; 14]	<0.001
Indexed LV mass (g/m2)	99 [78; 122]	68 [57; 86]	<0.001	79 [68; 100]	79 [66; 91]	105 [82; 127]	<0.001
LV hypertrophy	305 (50%)	15 (10%)	<0.001	5 (26%)	18 (18%)	274 (57%)	<0.001
Relative Wall Thickness	0.4 [0.3; 0.4]	0.4 [0.3; 0.5]	0.047	0.3 [0.3; 0.4]	0.4 [0.3; 0.4]	0.4 [0.3; 0.4]	0.314
Mass/volume ratio	1.6 [1.3; 1.9]	1.6 [1.3; 2.1]	0.012	1.2 [1.2; 1.6]	1.5 [1.2; 1.7]	1.6 [1.3; 2.0]	0.285
Indexed RA surface (cm^2^/m^2^)	10 [8; 11]	7 [6; 9]	<0.001	8 [8; 10]	8 [6; 9]	10 [8; 12]	<0.001
Mitral flow: E wave pic velocity (m/s)	98 [84; 111]	83 [71; 93]	<0.001	94 [87; 105]	88 [76; 105]	100 [87; 113]	<0.001
Mitral flow: A wave pic velocity (m/s)	56 [48; 67]	53 [44; 63]	0.002	59 [52; 66]	55 [50; 67]	57 [48; 68]	0.004
E wave deceleration time (ms)	168 [135; 205]	170 [140; 207]	0.526	168 [158; 202]	167 [132; 207]	167 [136; 201]	0.700
E/E' ratio	4.7 [4.0; 5.5]	4.0 [3.5; 4.8]	<0.001	4.8 [3.9; 5.3]	4.6 [3.9; 5.2]	4.8 [4.0; 5.6]	<0.001
E/E' ≥13	1 (0.2%)	0 (0%)	#	0 (0.00%)	0 (0.00%)	1 (0.21%)	1.000
E/E' ≥8	21 (4%)	0 (0%)	#	0 (0.00%)	1 (1.03%)	19 (3.99%)	0.369
E/A ratio	1.7 [1.4; 2.1]	1.6 [1.3; 1.9]	0.344	1.7 [1.4; 1.8]	1.5 [1.3; 1.9]	1.7 [1.4; 2.1]	0.292
Diastolic dysfunction			0.605				0.303
Normal LV filling pattern	583 (96%)	141 (95%)		19 (100%)	92 (93%)	464 (96%)	
Grade 1	25 (4%)	7 (5%)		0 (0%)	7 (7.14%)	19 (3.95%)	
Grade 2	1 (0.2%)	0 (0%)		0 (0%)	0 (0%)	0 (0%)	
Grade 3	0 (0%)	0 (0%)		0 (0%)	0 (0%)	0 (0%)	
Tricuspid regurgitant jet velocity m/s)	2.2 [1.9; 2.5]	2.1 [1.8; 2.3]	<0.001	2.0 [1.3; 2.3]	2.0 [1.5; 2.5]	2.2 [2.0; 2.5]	0.361
Systolic pulmonary arterial pressure (mmHg)	24 [19; 30]	21 [15; 26]	<0.001	22 [9; 25]	24 [16; 29]	24 [19; 30]	0.204
Pulmonary vascular resistances (WU)	1.3 [0.7; 1.9]	1.1 [0.9; 2.1]	0.595	0.9 [0.7; 2.0]	1.4 [0.7; 2.1]	1.2 [0.8; 1.8]	0.008
TRV >2,5 m/s	161 (28%)	21(16%)	0.001	4 (22.2%)	22 (23.7%)	132 (29.6%)	0.431
TRV >3 m/s (~pulmonary hypertension)	37 (6%)	0 (0%)	<0.001	2 (11.1%)	4 (4.30%)	26 (5.83%)	0.435
Valvular heart disease (mild or moderate)	8 (1.3%)	2 (1.3%)	0.774	0 (0%)	1 (1%)	7 (1.4%)	0.825
Regional wall motion abnormalities^*^	1 (0,4%)	0 (0%)	#	0 (0%)	0 (0%)	1 (0.4%)	#

*Quantitative value are expressed in median [Q1; Q3] and qualitative data are expressed in n (%)*.

* *This parameter has been assessed in 276 patients from Cameroon with available data*.

** *p-value adjusted for age and sex with country as a nested variable*.

LA, left auricular; LV, left ventricular; RA, right auricular;RV, right ventricular; TAPSE, tricuspid annulus plane systolic excursion; TRV, tricuspid regurgitant jet velocity.

# *non evaluable*.

**Figure 1:**
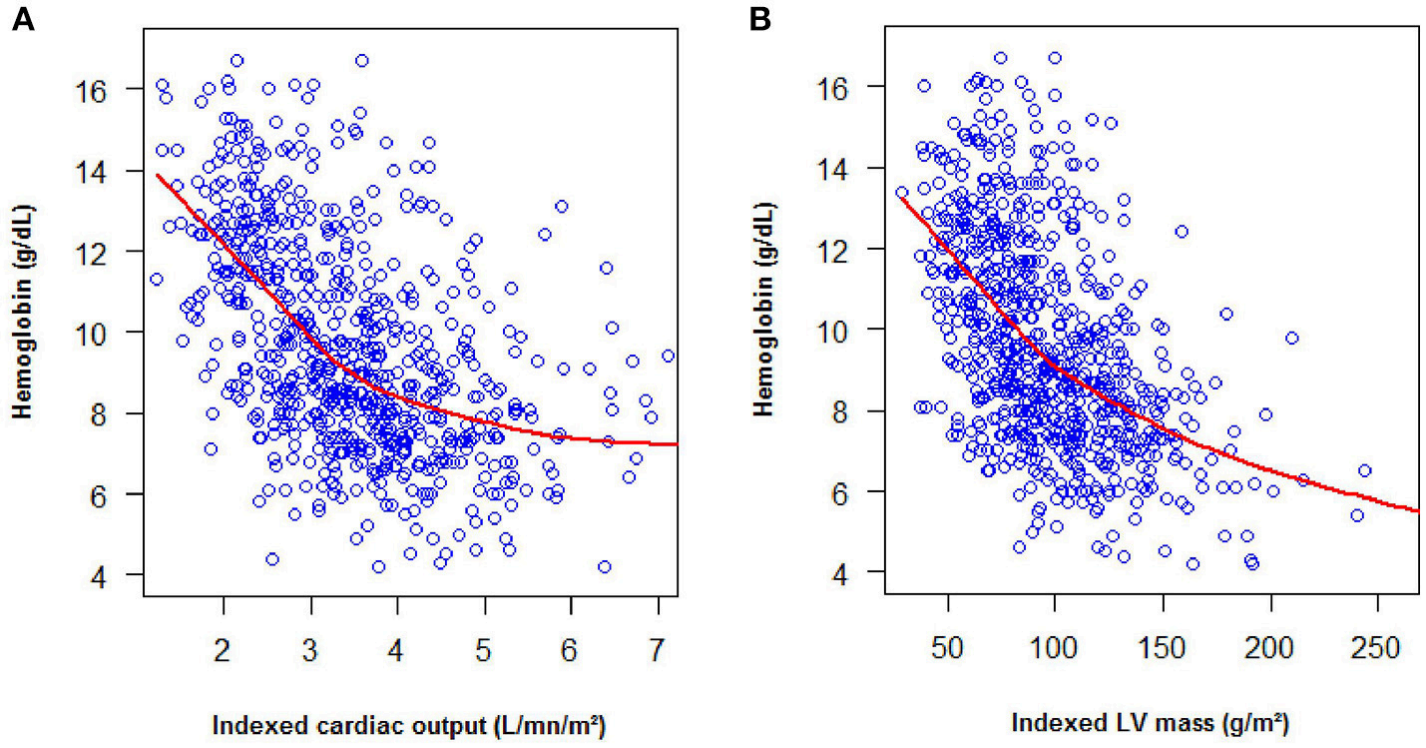
Evolution of cardiac output **(A)** and LV mass **(B)** depending on the level of hemoglobin.

#### Systolic function, diastolic function, and cardiac filling pressure

LV ejection fraction was similar in patients and in controls (67 [61;73] vs. 66 [60;71]%, *p* = 0.158). E wave deceleration time and diastolic dysfunction were not different between SCD patients and controls. E and A waves were higher in patients than in controls (respectively, 98 [84;111] vs. 83 [71;93] cm/s, *p* < 0.001 and 56 [48;67] vs. 53 [44;63], *p* = 0.002), but the E/A ratio was similar in both groups (*p* = 0.344). E/E' ratio was higher in patients than in controls (4.7 [4.0;5.5] vs. 4.0 [3.5;4.8], *p* < 0.001) but rarely reached a pathological level: E/E' was ≥8 in 21 (3.5%) patients vs. 0 controls (*p* = 1) and E/E' was ≥13 in 1 (0.2%) patient and 0 control, *p* = 1). Right ventricular function assessed by TAPSE (17 [15;20] vs. 14 [13;16], *p* < 0.001) and tricuspid annular Sdti (15 [13;17] vs. 13[12;15], *p* < 0.001) was higher in patients than in controls. Systemic resistances were lower in SCD patients (14 [11;17] vs. 20 [17;24] WU, *p* < 0.001].

#### Pulmonary pressure

TRV (2.2 [1.9;2.5] vs. 2.1 [1.8;2.3], *p* < 0.001) and sPAP (24 [19;30] vs. 21 [15;26], *p* < 0.001) were higher in patients than in controls. Conversely, systolic pulmonary resistances were not different between patients and controls (1.3 [0.7;1.9] vs. 1.1 [0.9;2.1] WU, *p* = 0.595). A TRV of more than 3 m/s was observed in 37 (6%) patients and 0 (0%) controls (*p* < 0.001).

#### Other echocardiographic features

Echocardiographic wall motion disorder was only evaluated in Cameroun and was observed in one (0.4%) patient vs. 0 control (*p* = 0.624). Valvular heart disease were equally prevalent in patients compared to controls [8 (1.3%) vs. 2 (1.3%), *p* = 0.774], all of them were of mild or moderate severity.

### Relationship between SCD-related organ complications and cardiac variables (Table 3)

In SCD patients, after adjustment for age, heart rate, mean arterial pressure, serum hemoglobin, phenotype of hemoglobin and country, cardiac chamber dilatation (LV, left and right atrium), LV mass (aOR = 1.7 [1.3;2.3]) and cardiac output (aOR = 1.3 [1;1.6]) were positively associated with SCD-related organ damages. After adjustment for the same variables, systemic vascular resistance (aOR = 0.7 [0.5;0.9]) was negatively associated with SCD-related organ damages. A sensibility analysis was conducted, which did not include osteonecrosis (that is typically associated with hyper-viscosity) among the other organ damages (that are typically associated with hyper-hemolysis), which displayed similar results (data not shown). A sensibility analysis was performed in the subgroup of SS/Sβ0 patients, showing similar results (Table S1).

**Table 3:** Multivariate associations between SCD-related extra-cardiac organ damage and echocardiographic measurements, adjusted for age, mean blood pressure, heart rate, hemoglobin, phenotype of hemoglobin and country.

		**Without organ damage *n =* 199**	**With organ damage *n =* 349**	**OR (IC 95%)**	***p-*value**	***n***
	Cardiac output index (L/m2)	3.4 ± 1.2	3.8 ± 1.1	1.3 (1;1.6)	0.010	515
	Systemic vascular resistances (WU)	16 ± 4	14 ± 6	0.7 (0.5;0.9)	<0.001	509
Left chambers	Indexed LV end-diastolic volume (mL/m2)	62 ± 17	67 ± 17	1.3 (1.1;1.7)	0.022	448
	Indexed LA surface (cm^2^/m^2^)	11 ± 3	13 ± 3	1.4 (1;1.9)	0.042	526
	Indexed LV mass (g/m^2^)	89 ± 35	109 ± 28	1.7 (1.3;2.3)	<0.001	528
	LV ejection fraction	68 ± 9	66 ± 8	0.9 (0.7;1.2)	0.371	530
Right chambers and pulmonary circulation	Indexed RV end diastolic diameter (mm/m^2^)	17 ± 4	19 ± 5	1.3 (0.9;1.7)	0.163	530
	Indexed RA surface (cm^2^/m^2^)	9 ± 3	10 ± 2	1.5 (1.2;2)	0.002	528
	Indexed TAPSE (mm/m^2^)	18 ± 4	18 ± 4	1.1 (0.9;1.4)	0.452	520
	Tricuspid regurgitant velocity (m/s)	2.0 ± 0.6	2.2 ± 0.5	1.1 (0.9;1.4)	0.442	490
	Systolic pulmonary arterial pressure (mmHg)	22 ± 11	25 ± 8	1.2 (1;1.5)	0.846	484
	Pulmonary vascular resistances (WU)	1.7 ± 0.9	1.4 ± 1.3	0.9 (0.7;1.3)	0.114	245
Diastolic function	E/E' ratio	4.8 ± 1.5	5.0 ± 1.4	1 (0.8;1.3)	0.967	522
	E/A ratio	1.8 ± 0.6	1.8 ± 0.6	0.9 (0.8;1.2)	0.546	524

*Quantitative variables are presented as mean ± SD*.

*Multilevel logistic regression adjusted for age, mean blood pressure, heart rate, hemoglobin, phenotype of hemoglobin and country*.

*Continuous variable were scaled in order to make OR comparable*.

*Extra-cardiac organ damage include microalbuminuria, osteonecrosis, leg ulcer, stroke, or priapism*.

## Discussion

In our sub-Saharan African study of teenagers and young adults, congestive heart failure and cardiac functional symptoms were extremely rare. We confirmed that SCD patients display a harmonious dilatation of cardiac atria and ventricles, as well as a high cardiac output, high cardiac work index with normal LVEF. However, unlike previous studies, we observed a very low frequency of pathological LV filling pressure in our population.

While “heart failure” has traditionally been considered common in adult SCD patients, there is no large screening study that describes the prevalence of congestive heart failure or cardiac functional symptoms. Although Sadchev and coll. showed that high E/E' ratio and TRV were associated to poor exercise capacity (15), recent studies have focused on echocardiographic parameters at a resting state. Systolic function is preserved in the majority of SCD patients (2, 13, 14, 16–19). Congestive heart failure has mainly been observed during acute complications of SCD such as vaso-occlusive crisis or acute chest syndrome. We could hypothesize that an acute drop of hemoglobin level leads to a cardiac work overload that may not be supported by the heart's contractile reserve (3, 4). However, pathological analysis of 52 hearts from deceased patients with sickle cell anemia found cardiac dilatation and hypertrophy with signs of congestive heart failure in 17/52 cases with neither coronary atherosclerosis, nor infarction (20). By contrast, in our population of teenagers and young adults at steady state, the prevalence of congestive heart failure was extremely low, not statistically different from null. It is possible that some cardiac failure-related symptoms were erroneously attributed to SCD (for instance, dyspnea attributed to anemia) but clinical acute congestive heart failure is unlikely to have been underdiagnosed. Nevertheless, some characteristics of our study may have led to underestimate the prevalence of heart failure. First, we did not assess the incidence of acute heart failure during vaso-occlusive crisis, acute chest syndrome or other causes of acute anemia. Second, the SCD population is younger in Africa than in the USA or Europe, and we cannot rule out an increased incidence of heart failure in case of prolonged survival, although the prevalence in the oldest patients (older than 40 years old) was not significantly higher; Third, the cross-sectional design of the study cannot rule out a survivor bias, that may be higher than in Europe and USA studies due to earlier death. Nonetheless, our study suggests that cardiac failure is rare feature in patients living with SCD in Africa.

In contrast, echocardiographic measures revealed a major cardiac remodeling in these patients with homogeneous dilatation and LV hypertrophy, in line with echocardiographic studies performed in SCD patients from other regions, as well as in patients with other causes of chronic anemia (1). We found no difference of prevalence of LV systolic or diastolic dysfunction between SCD patients and healthy controls using the usual echocardiographic definitions [LV ejection fraction <45%; E'l <10 cm/s or E's <8 cm/s (11)]. However, although they did not reach a pathological level, LV filling pressure (defined by E/E' ratio) were higher in patients than in controls which argues in favor of a subclinical cardiac dysfunction. Noteworthy, those findings are subject to criticism because echocardiographic diastolic dysfunction criteria are poorly validated in the case of high cardiac output (21). As other authors, we did not find any echocardiographic LV systolic dysfunction. As the LV ejection fraction is not the best measure to evaluate early LV systolic dysfunction, several authors have used longitudinal strain which assesses more accurately myocardial deformation. Most studies did not show any significant abnormality in LV strain (22–26) and only one study reported right ventricular lower strain in children with SCD compared to healthy children (24).

In theory, different mechanisms could lead to heart failure in SCD patients, including volume overload or myocardial ischemia. Volume overload is correlated with the degree of chronic anemia, especially when the hemoglobin is under 9 g/dL (Figure 1). This overload is probably responsible for heart failure in case of acute anemia. Noteworthy, Bahl et al. found that chronic severe anemia alone did not lead to cardiac disease (27). A few studies support the role of ischemia in SCD heart failure as the myocardial flow reserve is abnormal in SCD patients (4–6) but, as mentioned above, the occurrence of myocardial infarction is rare(5, 20). These findings are in accordance with the very low rate of regional wall motion abnormalities observed in our study. Rare myocardial infarctions are likely due to capillary obstruction whilst coronary arteries are usually normal (5, 28). Noteworthy, despite the African location of our study, the rate of valvular heart disease was lower than expected (29), possibly due to the penicillin prophylaxis that is recommended during childhood in SCD, thereby preventing the occurrence of rheumatic heart disease.

Regarding the pulmonary circulation, we found higher TRV in patients than in controls, which corroborates other large scale studies (13, 14). As demonstrated by Parent et al. (12) in an invasive hemodynamic study, the estimated high sPAP by echocardiography in SCD patients is mostly due to high cardiac output, and only rarely reflects a true pulmonary arterial hypertension (high pulmonary arterial resistances) or a cardiac dysfunction (post capillary pulmonary hypertension due to the rise of LV filling pressure). In our study, pulmonary vascular resistances assessed by echocardiography were similar in patients and controls while there was a clear decrease in systemic vascular resistances (30), suggesting a lack of pulmonary resistances adaptation.

Although we observed that SCD cardiac remodeling rarely went to pathological filling pressure and clinical symptoms of congestive heart failure, cardiac dilatation (LA, L,V, and RA surfaces) and LV mass were independently associated with the presence of SCD-related organ damages, including glomerulopathy, osteonecrosis, leg ulcers, priapism, and stroke. This association persisted after adjustment for major confounders (age, mean BP, heart rate, hemoglobin, sickle cell phenotype and country). Lower systemic vascular resistances were also independently associated with SCD-related organ damages. Surprisingly, TRV ratio was not associated with extra-cardiac organ damages in our cohort, whereas high TRV has been reported to be more frequent in patients with hyper-hemolysis phenotype, together with more frequent leg ulcers and priapism (31). However in another report of the CADRE study, we showed that this association between high TRV, leg ulcers, priapism and increased hemolysis markers was not clinically relevant in our African population (32). Nevertheless, high TRV has been consistently reported as a strong risk factor of mortality in SCD and is certainly worth monitoring as a global prognostic factor (14, 18).

The main strength of this study is to be the largest and the first multicentric cohort of SCD patients with cardiac evaluation in sub-Saharan Africa to date. Furthermore, patients were prospectively recruited irrespective of their symptoms, to avoid any referral bias, and both children (over 10 years) and adults of all SCD phenotypes were represented. However, we acknowledge some notable methodological limitations, especially the absence of Echo Core lab, which was impossible to set up in this multicentric African context and the absence of inter- and intra-reader variability calculation. Moreover, the cross-sectional design of the study does not allow inferring any temporality or causal link between cardiac abnormalities and extra-cardiac organ damage.

## Conclusion

Our results suggest that the major cardiac remodeling observed in the SCD exceptionally leads to clinical or echocardiographic heart failure in West and Central African patients with SCD but is associated with other organ damages. The prognostic value of cardiac measurements in sub-Saharan Africa will be better assessed during the follow-up of the patients. It will help identify non-invasive predictors of SCD-related organ damages and better tailor the monitoring and treatment of SCD patients in this resource constrained setting.

## Author contributions

XJ and IBD conceived the presented idea. AM and XJ designed the study. XJ encouraged AM to investigate and XJ supervised the findings of this work. AM wrote the manuscript with support from BR. AM and LO performed the analytic calculations. BR, XJ, SM, and MM contributed to the final version of the manuscript. BR and XJ supervised the project. AM, IBD, SK, RN, MD, DD, SD, ID-L, AT, IS, DC, GW, ID, BF, MS, AT, KB, GK, EA, CD, YT, GL, IK, and LO contributed to the implementation of the research, and collected the data. IBD, SK, RN, MD, DD, SD, ID-L, AT, IS, and DC supervised the study in the different countries. All authors discussed the results and contributed to the final manuscript.

### Conflict of interest statement

The authors declare that the research was conducted in the absence of any commercial or financial relationships that could be construed as a potential conflict of interest.
