# Supplementary material for: Subclinical Cardiac Dysfunction Is Associated With Extracardiac Organ Damages
Source: Front Med (Lausanne). 2018 Nov 20;5:323. doi: 10.3389/fmed.2018.00323 (PMC6262341; doi:10.3389/fmed.2018.00323)
Supplement: Supplementary file 1 [file Table_1.docx]

**Supplementary Material**

Table S1. Multivariate associations between SS/Sβ0-related extracardiac organ damage and echocardiographic measurements, adjusted for age, mean blood pressure, heart rate, hemoglobin and country.

| . | |  |  |  |  |  |
| --- | --- | --- | --- | --- | --- | --- |
|  |  | **without organ damage** | **with organ damage** | **OR** | **p-value** | **n** |
|  |  | **n=151** | **n=293** | **(IC 95%)** |  |  |
|  | **Cardiac output index (L/m2)** | 3.5 ± 1.0 | 3.9 ± 1.2 | 1.3 ( 1 ; 1.7 ) | 0.025 | 416 |
|  | **Systemic vascular resistances (WU)** | 15 ± 4 | 13 ± 5 | 0.6 ( 0.5 ; 0.8 ) | <0.001 | 411 |
| *Left chambers* | **Indexed LV end-diastolic volume (mL/m2)** | 65 ± 17 | 69 ± 18 | 1.5 ( 1.1 ; 2 ) | 0.011 | 345 |
|  | **Indexed LA surface (cm²/m²)** | 12 ± 3 | 13 ± 3 | 1.2 ( 0.9 ; 1.6 ) | 0.221 | 422 |
|  | **Indexed LV mass (g/m2)** | 93 ± 34 | 113 ± 28 | 1.7 ( 1.2 ; 2.3 ) | <0.001 | 424 |
|  | **LV ejection fraction** | 67 ± 9 | 65 ± 8 | 1 ( 0.7 ; 1.2 ) | 0.710 | 425 |
| *Right chambers and pulmonary circulation* | **Indexed RV end diastolic diameter (mm/m²)** | 17 ± 4 | 19 ± 5 | 1.3 ( 0.9 ; 1.8 ) | 0.182 | 425 |
|  | **Indexed RA surface (cm²/m²)** | 9 ± 3 | 11 ± 2 | 1.7 ( 1.3 ; 2.2 ) | <0.001 | 423 |
|  | **Indexed TAPSE (mm/m²)** | 17 ± 4 | 18 ± 4 | 1.1 ( 0.9 ; 1.5 ) | 0.301 | 418 |
|  | **Tricuspid regurgitant velocity (m/s)** | 2.1 ± 0.5 | 2.2 ± 0.5 | 1 ( 0.8 ; 1.3 ) | 0.921 | 392 |
|  | **Systolic pulmonary arterial pressure (mmHg)** | 22 ± 10 | 26 ± 8 | 1.1 ( 0.9 ; 1.5 ) | 0.400 | 387 |
|  | **Pulmonary vascular resistances (WU)** | 1.4 ± 0.7 | 1.3 ± 0.8 | 0.7 ( 0.4 ; 1.1 ) | 0.119 | 172 |
| *Diastolic function* | **E/E' ratio** | 4.9 ± 1.5 | 5 ± 1.4 | 1.1 ( 0.8 ; 1.3 ) | 0.630 | 419 |
|  | **E/A ratio** | 1.9 ± 0.5 | 1.8 ± 0.6 | 0.9 ( 0.7 ; 1.2 ) | 0.631 | 421 |
| Quantitative variables are presented as mean ± SD | | | | | | |
| Extra-cardiac organ damage include microalbuminuria, osteonecrosis, leg ulcer, stroke, or priapism | | | | | | |
